# L'accident ischémique cérébral chez le sujet jeune: à propos de 6 cas

**DOI:** 10.11604/pamj.2015.22.142.7609

**Published:** 2015-10-14

**Authors:** Naziha Khammassi, Yosra Ben Sassi, Asma Aloui, Youssef Kort, Haykel Abdelhedi, Ouahida Cherif

**Affiliations:** 1 Service de Médecine Interne, Hôpital Razi, La Manouba, Tunisie

**Keywords:** Accident ischémique cérébral, sujet jeune, diagnostic, étiologies, traitement, Transient ischemic attack, young adult, diagnosis, etiology, treatment

## Abstract

Les accidents ischémiques cérébraux (AIC) du sujet jeune se caractérisent par une panoplie d’étiologies différentes de celles des accidents vasculaires cérébraux (AVC) du sujet âgé d'où l'intérêt de bien creuser devant une telle atteinte à la recherche surtout d'une thrombophilie ou d'une cardiopathie emboligène. Cependant, il ne faut pas négliger une exposition de plus en plus accrue du sujet jeune à des facteurs de risque cardio-vasculaires tel le tabagisme qui accélère le processus d'athérosclérose, étiologie principale d'AVC tout âge confondu. Nous avons rétrospectivement collecté les données de six patients âgés de moins de 45 ans qui ont été hospitalisés dans notre service pour un AIC. La moyenne d’âge était de 35,3 et les hommes représentaient 16% de notre série. L'enquête étiologique a conclu à un syndrome des anticorps anti-phospholipides secondaire à un syndrome de Gougerot Sjögren chez l'un des patients, un déficit en protéine S chez deux patients et un syndrome de Sneddon chez un autre. Les causes d'AIC n'ont pas été identifiées dans les deux autres cas. Un traitement à base d'anti-vitamines K ou d'antiagrégants plaquettaires a été instauré en cas d’étiologie révélée.

## Introduction

L'accident ischémique cérébral (AIC) du sujet jeune de moins de 45 ans [1] s'authentifie à l'ensemble des AVC par sa présentation clinique et sa prise en charge initiale. En revanche les AIC du sujet jeune se distinguent de part leurs aspects étiologiques et les thérapeutiques qui s'en suivent et ils représentent 10% de l'ensemble des AVC quelque soit l’âge [2]. Il est à noter également que les AIC du sujet jeune posent un problème de diagnostic différentiel avec certaines pathologies aussi fréquentes dans cette tranche d’âge exigeant ainsi une enquête étiologique attentive. L’évolution et le pronostic sont en général meilleurs par rapport aux atteintes du sujet âgé, tout de même une meilleure prise en charge et surtout l’éviction des récidives, nécessite d'appréhender la bonne démarche diagnostique et thérapeutique.

## Méthodes

Nous rapportons six cas d'AIC diagnostiqués chez des sujets de moins de 45 ans dans le service de médecine interne de l'hôpital Razi dans la période s’étendant de 2010 à 2014. Les données ont été recueillies à partir des dossiers cliniques et pour chaque patient nous avons collecté: des éléments épidémiologiques: âge, sexe, antécédents personnels; des éléments cliniques: examen somatique complet à l'admission, notamment l'examen neurologique; des éléments biologiques: hémogramme, bilan inflammatoire, bilan d'hémostase, bilan lipidique, bilan immunologique, bilan de thrombophilie et le dosage de l'homocystéine; des éléments de l'imagerie: tomodensitométrie cérébrale, l’échographie tansthoracique et des troncs supra-aortiques.

## Résultats

### Population de l’étude

Nos résultats sont consignés dans des tableaux comme tel: les données épidémiologiques et cliniques, les données biologiques, les données radiologiques et les données immunologiques respectivement dans les Tableau 1, Tableau 2, Tableau 3, Tableau 4).

**Tableau 1:** Données épidémiologiques et cliniques

	Cas 1	Cas 2	Cas 3	Cas 4	Cas 5	Cas 6
Sexe	femme	femme	femme	femme	femme	homme
Age	29	40	42	44	39	14
Antécédents **personnels**	Rhumatisme Articulaire aigu	AIT Avortements spontanés à répétition	-	Convulsion fébrile	AIT	Rhumatisme articulaire aigu
Examen	Livédo réticulaire des extrémités+ mouvements anormaux	Déficit moteur et sensitif de l'hémicorps gauche	Hémiparésie gauche	Confusion	Hémiplégie gauche	Aphasie

AIT : accident ischémique transitoire

**Tableau 2:** Données biologiques et radiologiques

	Cas 1	Cas 2	Cas 3	Cas 4	Cas 5	Cas 6
NFS	Normale	Normale	Anémie ferriprive	Anémie ferriprive	Anémie ferriprive	Normale
Bilan d'hémostase	Normal	Normal	Normal	Normal	Normal	Normal
Bilan inflammatoire	Pas de SIB	Pas de SIB	Pas de SIB	Pas de SIB	Pas de SIB	Pas de SIB
Bilan lipidique	Normal	Normal	Normal	Normal	Normal	Normal
TDM cérébrale	Séquelles d'accident ischémique sylvien superficiel gauche et une atrophie cortico-sous corticale	Hypodensité cortico-sous corticale temporale gauche d'allure ischémique ancienne	Accident ischémique capsulo -thalamo -caudé	Séquelles ischémiques du territoire jonctionnel postérieur gauche	Hypodensité ischémique dans le territoire sylvien	Ischémie dans le territoire de la cérébrale moyenne droite

NFS : numération formule sanguine, SIB : syndrome inflammatoire biologique, TDM : tomodensitométrie

**Tableau 3:** Explorations cardio-vasculaires

	Cas 1	Cas 2	Cas 3	Cas 4	Cas 5	Cas 6
**ECG et holter rythmique**	Sans anomalies	Sans anomalies	Sans anomalies	Sans anomalies	Sans anomalies	Sans anomalies
**ETT**	Normale	Normale	Normale	Normale	Fuite mitrale minime Valve mitrale légèrement remaniée	Insuffisance mitrale minime
**ETSA**	Normale	Normale	Normale	Normale	Normale	Normale

ECG: électrocardiogramme, ETT: échographie cardiaque tansthoracique, ETSA: échographie des troncs supra-aortiques

**Tableau 4:** Données immunologiques

	Cas 1	Cas 2	Cas 3	Cas 4	Cas 5	Cas 6
**Bilan immunologique**	Sans anomalies	Anticorps anti-béta 2 glycoprotéine et AAN positif	Sans anomalies	AAN négatifs Anti DNA négatif Anti-phospholipides négatifs	AAN positif Anti DNA négatif Anti-phospholipides négatif	AAN négatifs Anti DNA négatif Anti-phospholipides négatifs
**Bilan de thrombophilie**	Sans anomalies	Sans anomalies	Déficit en protéine S	Déficit en protéine S	Sans anomalies	Sans anomalies
**Homo-cystéinémie**	Normale	Normale	Normale	Normale	Normale	Normale

AAN: anticorps antinucléaires

### Etiologies

L'enquête étiologique a conclu à un syndrome de Sneddon pour le premier cas devant l'association d'un livédo et d'une atrophie cortico-sous corticale (Tableau 1, Tableau 2). Pour le deuxième cas, le diagnostic de syndrome des antiphospholipides secondaire à un syndrome de Goujerot Sjögren a été retenu (Tableau 4). Le déficit en protéine S a été retenu comme étiologie de l'AIC pour le troisième et le quatrième cas (Tableau 4). Les différentes explorations n'ont pas permis de retenir une étiologie pour le cinquième et le sixième cas.

## Discussion

Age et sexe

La moyenne d’âge de nos patients était de 35,3 ans, presque identique à celle retrouvée dans la majorité des séries [3, 4]. Les hommes représentaient uniquement 16% de notre effectif avec une nette prédominance féminine. Ceci parait être en contraste avec la plupart des études récentes qui ont retrouvé une légère prédominance masculine. Dans l’étude qui a été menée par Cerrato et al, les hommes représentaient 58% de la population [3]. Un autre registre étudié par Kristensen et al rejoignait encore ces résultats avec un pourcentage de 59% de sujets masculins. Toutefois il est à noter qu'une étude de Bogousslavsky et al. a démontré le contraire avec un pourcentage de femmes beaucoup plus important (56%) [1]. En raison de notre faible effectif, cette apparente prédominance féminine est difficile à authentifier.

Démarche diagnostique

Une étape ultime après la survenue d'un AIC chez un sujet jeune consiste à affirmer ou à infirmer une dissection vasculaire dont la confirmation diagnostique est rendue aisée grâce aux moyens d'imagerie cérébrale. Une fois ce diagnostic récusé, la recherche d'une anomalie de la coagulation est justifiable. D'ailleurs ceci nous a permis de retrouver un déficit en protéine S chez deux de nos patients et de le retenir comme étiologie de l'accident cérébral en l'absence d'autres causes.

Topographie lésionnelle

Dans les six cas étudiés, l'ischémie cérébrale est survenue dans le territoire sylvien sans aucune atteinte de la région vertébro-basilaire. L’étude Cerrato et al a retrouvé un pourcentage de 52% d'ischémie dans le territoire sylvien contre 36% dans la région vertébro-basilaire [3].

Etiologies

Parmi les six cas étudiés dans notre service, les causes d'AIC n'ont pu être déterminées dans 33% des cas. Notre effectif, bien que faible, traduisait les données de la littérature. En effet dans la plupart des séries, l'enquête étiologique demeurait négative chez près de 30% des patients malgré un bilan dit exhaustif. Ce pourcentage pouvait aller à 50% dans certaines études [5]. De tels résultats reflétaient toute la difficulté à établir le diagnostic étiologique de l'accident cérébral chez le sujet jeune. En revanche certaines étiologies ressortaient comme les plus fréquentes dans la plupart des études. Ainsi les cardiopathies emboligènes suivies des dissections de l'aorte étaient identifiées comme les étiologies les plus courantes des AVC du sujet jeune [1, 4]. Toutefois ceci ne pourrait dissimuler la grande variabilité étiologique de ces accidents chez les jeunes patients, ce qui exige justement une discussion et une prise en charge individualisées et propres à chaque situation clinique. Tableau 5 montre les principales causes retrouvées dans la plupart des études. Il est justement remarquable que l'incidence de certaines étiologies diffère en fonction du type de l'enquête, la taille de l’échantillon et les moyens de diagnostics utilisés.

**Tableau 5:** Étiologies des AIC du sujet jeune dans d'autres études

Etiologies (%)	Razzaq et al 2002 [6]	Kristensen et al 1997 [7]	Bevan et al 2012 [8]	Adams et al 2007 [9]	Nencini et al 1988 [10]
**Athérosclérose**	22	12	31,2	17,6	22,3
**Maladies vasculaires non athéromateuses**	8,5	17	10,4	0	0
**Cardiopathies emboligènes**	11	35	35,4	17,4	22,2
**Thrombophilie**	3,4	8	16,7	0	0
**Migraine**	2,5	1	2,1	0	5,5
**Autres**	6,7	5	0	0	22
**Indéterminée**	45,8	21	4,2	34,3	22

Traitement

La prise en charge initiale des jeunes patients atteints d'AIC est semblable à celle des sujets âgés, par contre un traitement spécifique a pu être instauré chez trois de nos patients. Le patient chez qui on a diagnostiqué un syndrome des anticorps antiphospholipides a été mis sous antiagrégant plaquettaire, par contre ceux ayant un déficit en protéine S ont été mis sous anti-vitamines K avec une bonne évolution clinique et absence de récidive. Ainsi le traitement des AIC dépend de l’étiologie, si on arrive à l'identifier et il rejoint le traitement des AVC du sujet âgé dans le cas contraire.

## Conclusion

Les AIC du sujet jeune nécessitent une approche diagnostique individualisée du fait de la fréquence relative de pathologies sous jacentes, bien que l'athérosclérose demeure en tête de liste des étiologies tout âge confondu. L'imagerie cérébrale a tout son intérêt que ce soit pour établir un diagnostic topographique ou pour avoir une orientation étiologique en révélant certaines anomalies spécifiques. Tout de même elle ne dispense pas d'un bilan biologique visant à détecter surtout une anomalie de l'hémostase ou une dysimmunité ni d'explorations cardiaques à la recherche d'une cardiopathie emboligène. D'autres investigations peuvent être nécessaires en fonction du contexte clinique.
